# Supplementary material for: Brain-localized CD4 and CD8 T cells perform correlated random walks and not Levy walks
Source: F1000Res. 2023 Oct 3;12:87. Originally published 2023 Jan 23. [Version 2] doi: 10.12688/f1000research.129923.2 (PMC10556561; doi:10.12688/f1000research.129923.2)
Supplement: Peer review history

# Reviewer report: Reviewer response for version 2

**DOI:** 10.5256/f1000research.155847.r211618

**Published:** 2023-11-01

**Authors:** Masato S. Abe

**Affiliations:** 1 Doshisha University, Kyoto-fu, Japan

I think it has improved enough.

Is the work clearly and accurately presented and does it cite the current literature?

Yes

If applicable, is the statistical analysis and its interpretation appropriate?

Yes

Are all the source data underlying the results available to ensure full reproducibility?

Yes

Is the study design appropriate and is the work technically sound?

Yes

Are the conclusions drawn adequately supported by the results?

Yes

Are sufficient details of methods and analysis provided to allow replication by others?

Partly

Reviewer Expertise:

Movement ecology, complex systems

I confirm that I have read this submission and believe that I have an appropriate level of expertise to confirm that it is of an acceptable scientific standard.

# Reviewer report: Reviewer response for version 2

**DOI:** 10.5256/f1000research.155847.r211616

**Published:** 2023-10-11

**Authors:** Rob De Boer

**Affiliations:** 1 Theoretical Biology, Utrecht University, Utrecht, The Netherlands

I have read this last version.

Is the work clearly and accurately presented and does it cite the current literature?

Partly

If applicable, is the statistical analysis and its interpretation appropriate?

Yes

Are all the source data underlying the results available to ensure full reproducibility?

Yes

Is the study design appropriate and is the work technically sound?

Yes

Are the conclusions drawn adequately supported by the results?

Yes

Are sufficient details of methods and analysis provided to allow replication by others?

Yes

Reviewer Expertise:

Theoretical Immunology

I confirm that I have read this submission and believe that I have an appropriate level of expertise to confirm that it is of an acceptable scientific standard.

# Reviewer report: Reviewer response for version 2

**DOI:** 10.5256/f1000research.155847.r211617

**Published:** 2023-10-05

**Authors:** Jens Stein

**Affiliations:** 1 University of Fribourg, Fribourg, Switzerland

The manuscript is acceptable in its current form.

Is the work clearly and accurately presented and does it cite the current literature?

Yes

If applicable, is the statistical analysis and its interpretation appropriate?

Yes

Are all the source data underlying the results available to ensure full reproducibility?

Yes

Is the study design appropriate and is the work technically sound?

Yes

Are the conclusions drawn adequately supported by the results?

Yes

Are sufficient details of methods and analysis provided to allow replication by others?

Yes

Reviewer Expertise:

Mouse immunology

I confirm that I have read this submission and believe that I have an appropriate level of expertise to confirm that it is of an acceptable scientific standard.

# Reviewer report: Reviewer response for version 1

**DOI:** 10.5256/f1000research.142643.r187061

**Published:** 2023-08-18

**Authors:** Jens Stein

**Affiliations:** 1 University of Fribourg, Fribourg, Switzerland

In the accompanying manuscript, Patel and colleagues have performed an in-depth re-examination of search strategies executed by brain-localized T cells, as assessed by published intravital microscopy data sets. Using a rigorous analysis pipeline, the authors identify a correlated random walk (CRW) pattern being consistent with T cell migration in two separate brain infection models. This contradicts a previous study, which has claimed that brain-resident T cells undergo Levy walks (LW), i.e., long displacements between periods of short displacements. Somewhat astoundingly, the previous conclusion of LW motility in brain-localized T cells appears to be mainly due to simple tracking errors, in which unrelated tracks were stitched together or missing time points filled in by the analysis program, giving rise to unphysiologically long displacements. In sum, the authors provide solid evidence of CRW as main movement pattern of T cells, a common strategy irrespective of the organ analyzed.

 I have only some minor concerns since the main take-home message for the "intravital community" - that careful image analysis is required to draw correct conclusions - is well documented. The author’s analysis is also timely, since erroneous claims on cell motility patterns are often pervasive (since presented as novel concepts) and therefore important to correct.

 Minor concerns:

 The authors occasionally switch between the two infection models (Toxoplasma and Plasmodium), e.g., on p. 3. last paragraph, or p. 8, 3rd paragraph, which makes it difficult for the reader to follow the manuscript.

 The authors describe on p. 5 that tracks with only a single position were removed. In other publications (e.g., Mempel et al, Nature, 2003), a more stringent selection is made, i.e., tracks followed by less than 5 consecutive time points are dismissed. This more stringent practice is routinely used in many laboratories, since very short tracks typically outnumber medium - to - long tracks. Can the authors comment on this?

 The comparison of the total number of brain-localized T cells estimated by intravital imaging in relation to flow cytometry-based enumeration is interesting, and I agree that flow cytometry tends to underestimate the actual number of cells. Nonetheless, the authors should additionally consider that inflammation is often localized in certain foci, which are then recorded for intravital microscopy, whereas adjacent brain areas might be virtually free of infiltrating leukocytes.

Is the work clearly and accurately presented and does it cite the current literature?

Yes

If applicable, is the statistical analysis and its interpretation appropriate?

Yes

Are all the source data underlying the results available to ensure full reproducibility?

Yes

Is the study design appropriate and is the work technically sound?

Yes

Are the conclusions drawn adequately supported by the results?

Yes

Are sufficient details of methods and analysis provided to allow replication by others?

Yes

Reviewer Expertise:

Mouse immunology

I confirm that I have read this submission and believe that I have an appropriate level of expertise to confirm that it is of an acceptable scientific standard, however I have significant reservations, as outlined above.

# Reviewer report: Reviewer response for version 1

**DOI:** 10.5256/f1000research.142643.r187062

**Published:** 2023-08-17

**Authors:** Masato S. Abe

**Affiliations:** 1 Doshisha University, Kyoto-fu, Japan

In this study, the authors quantitatively investigated the movement patterns of brain localized T-cells to determine whether they are Lévy walk or Brownian walk. A new analysis of the data as a separate trajectory starting from the missing data shows that T. gondii-specific CD8 T-cells, contrary to previous studies, do not exhibit a Lévy walk, but rather a correlated random walk pattern, similar to other T-cells. The approach is based on a variety of rigorous methods, including fitting a generalized Pareto distribution to whole movement length distributions and a power-law distribution to the tails, and so on. The characterization of movement patterns is an important for understanding cells and other biological systems. Therefore, the findings give insights into these research areas. Still, I have some comments below.

 Major comments

- The definition of movement length appears to be unwritten, which is an important factor since the results can vary depending on the definition of movement length.
- Is there a possibility that long movement length in T. gondii-specific CD8 T-cells is underestimated by splitting a trajectory with missing data?
- In the analysis of movement patterns for some species (e.g., mammals), a truncated power law distribution is sometimes fitted. However, in this manuscript, a pure power law distribution for the tail of movement length distribution was used. Visually, it doesn't look like the tail of the distribution is cut off, so I don't think additional analysis is necessary, but what are your thoughts on analysis based on truncated power law for cell movement patterns?
- How to determine r_min is very important as Clauset et al. (2009) pointed out, so please write it down in detail. The authors wrote they used the KS-test, but I think KS-statistic (i.e., distance between two distributions) is correct.
- I feel that the Supplement Fig.3 is important. So, it should be in the main text.

Minor comments

- In the description for the mean and variance of the GP distribution, the range of k is needed as k < 1 for the mean and k < 1/2 for the variance.
- Whole manuscript: Levy -> Lévy
- Supplemental Table S1: Why does the estimation of simulated Brownian walker and CRW walker have the same values?

Is the work clearly and accurately presented and does it cite the current literature?

Yes

If applicable, is the statistical analysis and its interpretation appropriate?

Yes

Are all the source data underlying the results available to ensure full reproducibility?

Yes

Is the study design appropriate and is the work technically sound?

Yes

Are the conclusions drawn adequately supported by the results?

Yes

Are sufficient details of methods and analysis provided to allow replication by others?

Partly

Reviewer Expertise:

Movement ecology, complex systems

I confirm that I have read this submission and believe that I have an appropriate level of expertise to confirm that it is of an acceptable scientific standard, however I have significant reservations, as outlined above.

# Reviewer report: Reviewer response for version 1

**DOI:** 10.5256/f1000research.142643.r161399

**Published:** 2023-02-22

**Authors:** Rob De Boer

**Affiliations:** 1 Theoretical Biology, Utrecht University, Utrecht, The Netherlands

This is an interesting paper first analyzing T cell migration data in the brain during Plasmodium infection (previously published data from Ghazanfari et al 2021), and then re-analyzing similar data during Toxoplasma infection (previously published data from Harris et al 2012). The main result is that, in contrast to what Harris et al concluded, there is no evidence for Levy walks in the data. Overall the analysis is rigorous and the results are convincing.

 My major recommendation is to markedly improve the style of writing. The presentation is quite unclear in places. Several sentences are written very poorly. The presence and absence of "the", "an" and "a" and commas seems pretty random. The senior author should be able to improve this. Otherwise seek help from a native English speaker. At the end, I will give a number of examples, but there are more sentences and paragraphs that need to be improved linguistically.

 Secondly, the authors use both track-based analysis and cell-based analyses. Joost Beltman in his review [Ref 7] indeed emphasized the importance of this, calling it track-based to step-based. It would be good in this paper to clearly make this difference, as the previous errors of Harris et al largely seem to be a consequence of their -sometimes improper- track based analysis.

 Page 7: In the first paragraph of the results you fail to tell the reader this is about Plasmodium data, and what PbA is (we apparently have to remember that from the Methods). Very confusing because you did tell us you will be using two data sets: which one is this section about?

 Page 8: A previous pioneering study proposed that brain-localized CD8 T cells perform generalized LWs characterized by rare but long displacements [Ref. 2, Figure 1B (iv)].

 => Now you are discussing Toxoplasma data (without telling us) and the next paragraph you switch back to Plasmodium again. Please make more clear which data set is being discussed.  Actually, because both have been published before you could treat them more equally.

 Page 8: Interestingly, 100-fold lower numbers were calculated for brain-localized T cells obtained using flow cytometry.  

 => So?  What do we need to make of this sentence here?

 Page 8: We first calculated the MSD for brain-localized Plasmodium-specific CD4 and CD8 T cell trajectories (equation (1)).

 => Track based?

 Page 8: We then analyzed the cumulative distribution of movement lengths which was similar for CD4 and CD8 T cells;

 => Step based?

 Page 8: To ensure that data sub-selection in the tail analysis did not bias the results, 

 => Explain, or refer to the Methods for an explanation. 

 Page 9: In our analyses, we found that brain- localized CD4 and CD8 T cells perform Brownian-type (correlated random) walks which contradict a previous result suggesting that movement patterns of CD8 T cells in the brain are consistent with generalized LWs.

 => In our analyses, we found that brain- localized CD4 and CD8 T cells DURING PLASMODIUM INFECTION perform Brownian-type (correlated random) walks which contradict a previous result suggesting that movement patterns of CD8 T cells in the brain DURING TOXOPLASMA INFECTION are consistent with generalized LWs.

 => Is that a contradiction or a difference?

 Page 11: Here we rigorously analyzed data from novel experiments imaging movement of Plasmodium-specific CD4 and CD8 T cells in brains of PbA-infected mice (Figure 1). 

 => Not novel: previously published.  See my remark above about treating the data more equally.

 Page 11: details slightly varied whether we compared instantaneous or per cell average speeds and turning angles

 => Is that a Step-based vs Track-based difference?

 Page 11: Calculating the number of T cells in the whole brain from imaging only 0.002% of its total volume may be problematic as T cells tend to leave and enter the imaging area. Using the number of T cells identified per only one time frame did not dramatically change the estimate of the total number of cells in the brain (results not shown).

 => Isn't that a Step-based vs Track-based difference?

 Page 12: Given the ability of T cells in tissues to do a persistent walk in a given direction (cell “inertia”

 => Is that an "ability" to a cellular constraint, i.e., a necessity?

 Page 12: the rule for T cell movements in tissues rather than THE exception.

 Page 13: that GP distribution => that the generalized Pareto distribution

 Page 13: Using vMF distribution => Using the von Moses-Fisher distribution

 => Both were only defined in the Methods!

 Page 13: such collaborations should be explicitly supported by funding agencies.

 => Is that really how you prefer to end, or are your scientific results the take home?

 ------

 Examples of improving linguistics:

 For survival of the organism, T cells must efficiently control pathogens invading different peripheral tissues but whether such control (and lack of thereof) is achieved by utilizing different movement strategies remains poorly understood. 

 => Please split sentence, e.g.,

 For survival of the organism, T cells must efficiently control pathogens invading different peripheral tissues. Whether or not such control is achieved by utilizing different movement strategies in different tissues remains poorly understood. 

 <= Moreover: how can a "lack of control" be achieved?

 Different agents starting with large animals such as tigers and wolves and ending with individual cells such as T lymphocytes typically search for resources/targets 

 => The sentence actually says that agents start to search with large animals and end to search with cells.

 In actual data, agents rarely show MSD to change linearly with time

 => Agents don't show MSD: In actual data the MSD is hardly ever linear

 In total, data from five movies were analyzed: three movies for 6.5 days post-infection and two movies for 7 days post-infection. 

 => so you have 3 movies spanning the first 6.5 days of infection and 2 movies for a week?  That is what the sentence says!  How long do these movies take actually?

 The constancy/decrease in CD4 T cell numbers is not fully consistent with flow cytometry data on kinetics of brain-localized endogenous T cell response to PbA.

 => Unclear. Later I read that the number of cells is not consistent.  What is "constancy/decrease?"

 In addition to the difference in the number between CD4 and CD8 T cells, we found

 => In addition to finding many more CD8 than CD4 T cells, we found

 Visual inspection of the five movies did not reveal any long displacements by T cells; however, there were some visible cell-like objects moving rapidly 

 => Omit the visible, the inspection was already visual

 Interestingly, fitting Furth equation to the MSD plots suggested much shorter persistence time of T cells of 1 min

 => Interestingly, fitting THE Furth equation to the MSD plots suggested A much shorter persistence time of T cells of 1 min

 However, in all cases, when simulated movements were non-Brownian, we found that GP fits indicated infinite variance (and sometimes mean) suggesting that this approach allows to detect deviations from Brownian walks. 

 => When simulated movements were non-Brownian, we found that THE GP fits always had an infinite variance (and sometimes mean), suggesting that this approach allows one to detect deviations from Brownian walks. 

 => And, what is this approach: the variance?

 One could expect that T cells should utilize different movement strategies when responding to diverse pathogens and migrating to different lymphoid or non-lymphoid (peripheral) tissues.

 => One could expect that T cells utilize different movement strategies when responding to diverse pathogens and when migrating to different lymphoid or non-lymphoid (peripheral) tissues.

 => could and should is double

 Given that in intravital imaging experiments it is typically hard to follow individual T cells for longer than 15-20 time frames, characterizing heterogeneity of movement of individual cells in vivo remains difficult (but see Ref. 14).

 => Difficult sentence 

 While imaging frequency used in the study by Ghazanfari et al.27 (30 sec) was sufficient to accurately detect movement of brain-localized T cells, it was clearly insufficient to better understand movement patterns of T cells in the vasculature, and as these cells move from the blood vessels into the parenchymal tissues. 

 => While THE imaging frequency used in the study by Ghazanfari et al.27 (30 sec) was sufficient to accurately detect movement of brain-localized T cells, it was clearly insufficient to better understand movement patterns of T cells in the vasculature, and as these cells move from the blood vessels into the parenchymal tissues. 

 => it remains unclear what the ", and as these cells move" means

Is the work clearly and accurately presented and does it cite the current literature?

Partly

If applicable, is the statistical analysis and its interpretation appropriate?

Yes

Are all the source data underlying the results available to ensure full reproducibility?

Yes

Is the study design appropriate and is the work technically sound?

Yes

Are the conclusions drawn adequately supported by the results?

Yes

Are sufficient details of methods and analysis provided to allow replication by others?

Yes

Reviewer Expertise:

Theoretical Immunology

I confirm that I have read this submission and believe that I have an appropriate level of expertise to confirm that it is of an acceptable scientific standard, however I have significant reservations, as outlined above.
